# Trace Elements in Home-Processed Food Obtained from Unconventional Animals

**DOI:** 10.3390/life10050075

**Published:** 2020-05-23

**Authors:** Emilio Carpenè, Giulia Andreani, Enea Ferlizza, Simonetta Menotta, Giorgio Fedrizzi, Gloria Isani

**Affiliations:** 1 Department of Veterinary Medical Sciences, Alma Mater Studiorum, University of Bologna, via Tolara di sopra 50, 40064 Ozzano Emilia (BO), Italy; emilio.carpene@unibo.it (E.C.); gloria.isani@unibo.it (G.I.); 2 Department of Experimental, Diagnostic and Specialty Medicine, Alma Mater Studiorum, University of Bologna, via Belmeloro 8, 40126 Bologna, Italy; enea.ferlizza2@unibo.it; 3 Istituto Zooprofilattico Sperimentale della Lombardia e dell’Emilia Romagna, Chemical Department, via P. Fiorini 5, 40127 Bologna, Italy; simonetta.menotta@izsler.it (S.M.); giorgio.fedrizzi@izsler.it (G.F.)

**Keywords:** metal intake, essential trace elements, non-essential trace elements, iron, lead, cadmium, home-processed food

## Abstract

Wild animals have been used as food since ancient times and, currently, the consumption of unconventional animals is increasing worldwide. The process of cooking meat using traditional recipes includes a variety of ingredients, which can influence the total metal intake from the diet. In this study, the concentrations of eight essential (Fe, Zn, Cu, Mn, Se, Ni, Mo, and Co) and six non-essential (Pb, Cd, Hg, Al, As, and Cr) trace elements were determined in home-processed food obtained from snails and from three common species of game animals (woodcock, pheasant, and hare), seasoned with anchovies, mushrooms, and different vegetables using inductively coupled plasma mass spectrometry (ICP-MS). In general, Fe was the most abundant trace element, ranging from 18 ± 8 µg/g in pheasant to 99 ± 76 µg/g in snail, and Co was the least abundant, ranging from 0.007 ± 0.003 µg/g in hare to 0.093 ± 0.048 µg/g in snail. Regarding the non-essential trace elements, Pb concentrations showed wide variations, reaching a concentration of 17.30 µg/g in hare, while Cd concentrations were higher in snail, ranging from 0.18 to 0.46 µg/g. These alternative food sources can offer an important contribution to the human nutritional requirements of essential trace elements, in particular of Fe. The high concentrations of Pb and Cd present in some samples should be considered as potentially dangerous for the consumers.

## 1. Introduction

Human diets include a myriad of animal sources, which vary widely in different countries. Wild animals have been used as food since ancient times and, currently, the consumption of unconventional animals from different taxa is increasing worldwide. Insects [1], mollusks [2] and vertebrates, ranging from fish to mammals [3], in particular game animals [4], are those most commonly used. The consumption of unconventional and exotic animals evokes strong emotions and controversy, reflecting the cultural background of the different societies [5]. Living in different biota, these animals represent an important source of nutrients, including proteins with high biological value and noticeable concentrations of essential trace elements, in particular Fe, Zn, Cu, Mn, Mo, Co, and Se. These elements are involved in cell metabolism and regulation, performing different biochemical functions which can be summarized as: (a) participating in redox reactions (Fe, Cu, Mn, Mo, and Co); (b) acting as enzyme cofactors (Fe, Zn, Cu, Mn, Co, and Mo); (c) acting directly or indirectly as antioxidants (Se, Zn, Cu, and Mn); and (d) participating in cell signaling processes (Zn) [6]. Consequently, the concentrations of these trace elements are finely regulated by sophisticated homeostatic mechanisms aimed at both ensuring their availability and at contrasting a harmful excessive intake, in particular for redox-active elements. At the same time, some non-essential trace elements (Pb, Cd, Hg, Al, and As) are also present in food and are considered only for their negative effects on human health [7].

The nutrient quality of food can be altered by different methods of food processing such as steaming or oven cooking. In particular, the loss of trace elements may be caused by their outflow from the food during cooking [1,3]. Similarly, bioaccessibility (i.e., the amount of trace elements available for intestinal absorption) can be modified by cooking, with emphasis on the reduction of non-essential element bioaccessibility, as has been reported in seafood [8]. The cooking process could vary from the simple grilling of meat to sophisticated recipes, which include a variety of ingredients influencing the total metal intake.

Many unconventional animals are traditionally and locally used as a source of food, however, their contribution to the dietary intake of trace elements has not yet received sufficient attention. In this study, the concentrations of eight essential (Fe, Zn, Cu, Mn, Se, Ni, Mo, and Co) and six non-essential (Pb, Cd, Hg, Al, As, and Cr) trace elements were determined in processed food obtained from snails and from three species of game animals (woodcock, pheasant, and hare), which are among the most commonly consumed in Italy [9]. The samples analyzed were obtained after home processing and cooking following traditional Italian recipes in order to obtain information regarding the “true” trace element intake from food after home-cooked preparations. Owing to the rich seasoning of these dishes, which included anchovies, mushrooms, and different vegetables, trace element concentrations were also determined in these ingredients. The contribution of Fe, Zn, and Cu to human nutritional requirements and the health risks due to the ingestion of Pb and Cd are also discussed.

## 2. Results

The concentrations of essential trace elements are reported in Table 1, Table 2, Table 3, Table 4, Table 5 and Table 6, according to their abundance in the samples analyzed. Data are reported in Table 1 for snails, in Table 2 for woodcock, in Table 3 for pheasant, in Table 4 for hare, in Table 5 for anchovies, and in Table 6 for mushrooms. The percentages of adequate dietary intake (AI) for the most relevant essential trace elements (Fe, Zn, Cu, Mn, Se, and Co) due to the consumption of 100 g of the processed meats are reported in Table 7.

Due to the ease of sampling, the snails were also analyzed as raw uncooked samples (Table S1). Trace element concentrations in the seasonings are reported in Supplementary Table S2. Moreover, the correlation among trace element concentrations in processed food was determined and the results are reported in Supplementary Table S3. Many statistically significant correlations were found; however, their significance was not in the scope of the present study. In general, Fe was the most abundant while Mo and Co were the least abundant essential trace elements. The non-essential trace elements are reported based on their abundance and toxicological hazard; Pb and Cd were the most represented in the samples analyzed.

### 2.1. Essential Trace Elements

Of the essential trace elements, Fe, Cu, Zn, and Mn were present in detectable concentrations in all the samples, while Ni, Mo, Se, and Co were below the limit of quantification (<LOQ) in a few samples of seasonings and mushrooms. With the exception of Se, the essential trace elements presented higher concentrations in both the raw and the processed samples of snails; in particular, the viscera showed the highest concentrations of trace elements.

Iron was the most abundant element, reaching its highest concentrations in snails: 99.64 ± 76.47 µg/g in cooked snails and 334.6 ± 402.3 µg/g in the viscera of raw snails. Regarding the other samples, Fe concentrations followed this decreasing order: woodcock (63.49 ± 23.80 µg/g), hare (33.74 ± 16.40 µg/g), and pheasant (18.76 ± 8.42 µg/g, significantly lower than woodcock and snails, *p* < 0.05). In the mushrooms and seasonings, the levels were significantly lower (*p* < 0.05) than in the viscera of raw snails.

Zinc concentrations presented a narrow range of concentrations in all the samples analyzed, ranging from 10.44 ± 1.35 µg/g in woodcock to 23.90 ± 6.539 µg/g in snails. Copper and Mn concentrations had a pattern similar to Fe. The lowest mean Cu concentration was detected in pheasant (1.373 ± 1.097 µg/g) and the highest in snails (75.13 ± 43.03 µg/g), significantly higher than in hare and pheasant samples (*p* < 0.05). Manganese concentrations ranged from 0.678 ± 0.419 µg/g in pheasant to 25.49 ± 12.55 µg/g in snails, significantly higher than in pheasant and hare samples (*p* < 0.05). In the seasonings, the concentrations of Cu and Mn were generally lower than in the processed meat.

Of the other trace elements, Se presented the highest concentrations in processed meat, with a maximum value of 0.583 ± 0.154 µg/g in woodcock and a minimum value of 0.215 ± 0.148 µg/g in hare. In snails, the Se concentration was lower and, in the raw snails, it was significantly lower in the foot than in the viscera (*p* < 0.05). Selenium was also abundant in commercial anchovies, with a mean concentration of 0.553 ± 0.027 µg/g. Vegetable seasonings showed Se concentrations close to the LOQ, while, in mushrooms, the values ranged from 0.014 µg/g to 0.855 µg/g. Of the cooked meat, the highest Ni concentrations were found in snails while, in the other samples, the metal content was lower. The viscera of raw snails had a Ni concentration significantly higher than the foot (*p* < 0.05). The highest Mo concentrations were measured in seasonings; however, high concentrations were also found in cooked snails (0.331 ± 0.049 µg/g), which were significantly higher (*p* < 0.05) than in hare. Finally, Co was the essential trace element present in the lowest concentrations in all the samples analyzed, ranging from 0.007 ± 0.003 µg/g in hare to 0.093 ± 0.048 µg/g in cooked snails; the latter value was significantly higher than those determined in pheasant and hare (*p* < 0.05).

The percentages of the adequate intake (AI) calculated for a consumption of 100 g of processed meat varied according to the species and the element analyzed (Table 7). The highest intake of Fe was obtained for snail (90% of AI) and the lowest for pheasant (17% of AI). Remarkably high Cu intakes were observed for snail, reaching percentages over 450% and 550% of the AI set for men and women, respectively. Snails also showed the highest intake of Mn, covering 83% of the AI, while very low intakes of this element can be obtained from the processed meat obtained from woodcock, pheasant, and hare. Regarding Zn, the samples analyzed showed percentages of AI between 9% for pheasant to 19% for snails.

### 2.2. Non-Essential Trace Elements

Lead concentrations were, in general, higher in the game animals than in the snails and mushrooms. The values also showed wide variations among the samples obtained from the same species, ranging from 0.01 to 0.470 µg/g in pheasant, from 0.407 to 2.421 µg/g in woodcock, and from 0.019 to 17.30 µg/g in hare. In addition, in raw snail viscera, Pb concentrations were significantly (*p* < 0.01) different between the two sampling sites of the Quaderna Valley.

Cadmium concentrations were significantly higher (*p* < 0.05) in the processed snails than in pheasant and hare, ranging from 0.180 to 0.460 µg/g. In woodcock, the Cd levels were lower than in snails, but significantly (*p* < 0.05) higher than in hare, reaching a mean value of 0.141 ± 0.086 µg/g.

Mercury was found in relevant concentrations in only one sample of mushrooms (M1, 0.522 µg/g) and in one sample of pheasant preparation seasoned with mushrooms M1 (0.179 µg/g). In addition, in woodcock preparations seasoned with anchovies, the Hg concentrations varied from 0.043 to 0.069 µg/g while, in the anchovy fillets, it varied from 0.121 to 0.143 µg/g.

Aluminum presented the highest concentrations compared to the other non-essential trace elements in all the samples analyzed, with the sole exception of anchovies. Aluminum concentrations varied widely from 1.464 ± 1.167 µg/g in pheasant to 41.11 ± 52.56 µg/g in snails. Very low concentrations of As were detected in the samples analyzed, with the exception of anchovies (2.693 ± 1.567 µg/g) and woodcock (0.528 ± 0.243 µg/g), as the concentrations in woodcock were significantly (*p* < 0.05) higher than those detected in pheasant and hare preparations. Chromium was present at detectable concentrations in all the samples analyzed, with the highest values in snails (0.339 ± 0.245 µg/g), followed by woodcock, mushrooms, hare, and pheasant.

## 3. Discussion

The home-processed food analyzed in this study could be considered an interesting and alternative source of essential trace elements, such as Fe, Zn, Cu, Mn, and Se. Unconventional animals, unlike farm animals, are free-ranging organisms and the chemical composition of their meat can be affected by several factors, including diet, environment, and animal lifestyle [12]. In addition, game animals present variable Pb contamination of their meat, due to the presence of Pb gunshot.

### 3.1. Essential Trace Elements

Of the samples analyzed, the highest Fe, Zn, Cu, Mn, and Mo concentrations were detected in snails. The concentrations in raw snail samples were in the range of those determined by other authors in *Helix pomatia* [13] and in *Cantareus aspersus* [14], but higher than those reported in the giant African snail *Achatina achatina* [15]. It is well known that land snails are able to accumulate high concentrations of trace elements, particularly Cu, due to the presence of a specific Cu-metallothionein isoform, which can act as a metal donor for the synthesis of hemocyanin [16]. This copper-containing protein is the oxygen carrier in the hemolymph of many mollusks [17]. Interestingly, a biochemical explanation is still lacking regarding the high concentrations of Fe and Mn. In particular, in snails, Fe is not required for hemoglobin, due to the presence of the respiratory protein hemocyanin, and the use of myoglobin seems to be limited to the radular muscles [18]. Regarding Mn, its high concentrations could be related to shell formation. In fact, it has been reported in freshwater bivalves that this trace element can substitute Ca in aragonite crystals [19].

In terms of processed vertebrate meat, higher concentrations of Fe, Mn, and Se were found in woodcock preparations, while the hare samples contained higher concentrations of Cu. The data obtained are in the range of those reported by other authors. Muscular Cu and Fe levels are related to the functional and biochemical characteristics of different fiber types. In woodcock and hare, the red color of the oxidative muscle fibers is due to the high levels of Fe and Cu, which are contained in hemoproteins (myoglobin, cytochromes) and cytochrome-c-oxidase, respectively [20]. Moreover, the pectoralis myosin of woodcock is unique due to a specific myosin heavy chain [21]. Pheasant preparations contained lower concentrations of Fe and Cu due to the high percentage of white pectoral muscle in the meat used for the preparation. Red muscles are rich in mitochondria, and these organelles are the main source of reactive oxygen species (ROS; particularly superoxide radical) in the cell. Antioxidant systems handling these harmful oxygen species are needed to prevent oxidative stress; superoxide dismutase, catalase, and glutathione peroxidase are the most active detoxifying enzymes metabolizing superoxide radicals and hydrogen peroxide. In particular, the high Mn concentrations detected in processed vertebrate meat are linked to the presence of mitochondrial manganese superoxide dismutase (MnSOD) acting as the chief ROS scavenging enzyme in the cell [22].

The homogeneity of Zn concentrations in the samples analyzed is due to the essential role of Zn in a wide range of biochemical systems shared by the majority of living organisms [23,24]. Consequently, Zn levels in tissues are maintained constant by means of homeostatic mechanisms, including specific membrane transporters and metallothioneins [25], and this is mirrored in the narrow range of Zn concentrations reported in the livers and kidneys of different organisms from invertebrates to mammals [2].

Nickel, Mo, and Co presented one-to-two orders of magnitude lower concentrations due to their limited involvement in the biochemical processes.

### 3.2. Non-Essential Trace Elements

The Pb and Cd concentrations determined in the raw foot of snails are low and of the same order of magnitude as those reported in raw meat samples of *H. pomatia* from Poland [26]. Ziomek et al. [26] reported that the cooking process likely increased the Cd content in snail meat. In the present study, it was not possible to compare metal concentrations in raw and cooked samples; conversely, it was reported that the metal concentrations were affected by the sampling site. Of interest are the concentrations of Al and Cr detected in snails, which are higher than those measured in all the other samples.

It is known that the wide variability of Pb concentrations in samples from game animals is due to the impossibility of removing all Pb gunshots or, alternatively, removing the contaminated meat [27,28]. Accordingly, a wide range of Pb concentrations has been reported in the muscles of game animals containing Pb gunshots, depending on the distance from the wound channel [29]. The Pb concentrations determined in this study, even though widely variable between 0.01 and 17.3 µg/g, were similar to or higher than the values reported in meat from game animals in Italy [30,31]. The lower Pb concentrations determined in the pheasant samples were probably due to the easier removal of Pb pellets in the white meat with respect to those present in the red meat of woodcock and hare. Different results were previously reported by Ertl et al. [32]. The authors analyzed the concentrations of Pb in the muscles of game animals in Austria, and pheasants were the most affected by Pb contamination, containing a mean metal concentration as high as 125 µg/g wet weight. All the samples of snails and the majority of the samples of the game animals included in this study, with the exception of five pheasant and three hare samples, had Pb concentrations higher than the maximum admissible level (ML) of 0.1 mg/kg established by the Commission Regulation (EC) No 1881/2006 [33]. Accordingly, it has been reported by Pain et al. [31] that an elevated proportion of tissues from both cooked and raw gamebirds had Pb concentrations exceeding the ML, even 10 fold, with a few samples exceeding the ML 100 fold [34]. On the contrary, in four pheasant samples (P1, P5, P6, and P7) and in one hare sample (H1), very low Pb concentrations were observed. These values, in the range of 0.01–0.03 µg/g, were similar to those reported by Gonzales-Weller et al. [32] in the meat of farmed animals and raised less concern [35].

The richness of the non-essential trace elements Cd, Al, As, and Hg in the woodcock pâtè could be considered a paradigm of how the different ingredients used for dish preparations, including seasonings, could influence the total metal intake. Therefore, in addition to raw material, it is also essential to determine the metal concentrations in processed food. In this case, Cd and Al derived from the woodcock viscera included in the food preparation while As and Hg came from the anchovies. It is well known that in vertebrates the liver and the kidney are the target organs of Cd accumulation due to the induction of metallothioneins [36,37,38]. In adult woodcock, a mean Cd concentration as high as 15.7 µg/g had previously been reported in the kidney, deriving from the woodcock diet based mainly on earthworms [39]. On the other hand, the presence of low, but not negligible, concentrations of Hg were due to the inclusion of anchovies during the preparation of the woodcock pâtè. This non-essential trace element is commonly found in fish, for the most part in the highly bioaccessible form methylmercury (MeHg) [40].

### 3.3. Benefit-Risk Balance

Assessment of the benefit–risk balance is an essential issue when evaluating the nutritional quality of food. Meat has been consumed for thousands of years and is considered an important source of proteins, vitamins, and essential trace elements. In the following paragraphs, the most relevant issues related to the samples analyzed in the present study will be discussed.

It has been estimated that more than two billion individuals from both developing and industrialized countries suffer from Fe deficiency, with the emphasis on young women [41]. At the same time, it has been well recognized that the distribution between heme and non-heme Fe is more important in determining the body metal status than the total dietary Fe intake [42]. Moreover, meat consumption is positively associated with a higher serum ferritin concentration [43] due to the high content of heme Fe, especially in red meat [44]. Snail and woodcock processed meat contains the highest Fe concentrations; considering the mean Fe values of 99.6 µg/g for snail and 63.5 µg/g for woodcock and assuming a consumption of 100 g of food, a total Fe intake of 9.96 mg and 6.35 mg, respectively, is obtained. These values represent 90.5% and 57.7%, respectively, of the population reference intake (PRI) of 11 mg/day reported by EFSAfor Fe [45]; therefore, snails could be considered a tasty and nutritious food, which could help reduce the anemia caused by a lack of Fe in the diet, in particular in developing countries. On the other hand, the high concentration and bioavailability of Fe in red meat could represent a risk for people suffering from cardiovascular diseases (CVDs). Several studies have examined the association between processed red meat consumption and the risk of CVDs, in particular, myocardial infarction. Different hypotheses, including the presence of heme Fe as a possible source of oxidative stress, have been reported to explain this association [46]. However, recent findings from the EPIC-Heidelberg (European Prospective Investigation into Cancer and Nutrition-Heidelberg) Study do not support the hypothesis that an increased Fe intake represents a mechanistic link between meat consumption and CVD risk because a high Fe status can also be associated with other physiological or pathological risk factors, including older age, male gender, obesity and inflammation [47]. More studies are needed on this challenging topic.

Snail processed meat contains high Cu concentrations. Considering the mean Cu level of 75.1 µg/g and assuming a consumption of 100 g of food, a total metal intake of 7.51 mg is obtained. This value represents 468% of the AIof 1.6 mg/day reported by EFSA for Cu in adult men. An even worse situation occurs for women, as the value represents 550% of the AI of 1.3 mg/d [48]. In both cases, the tolerable upper intake level (UL) of 5 mg/d set by EFSA for Cu is exceeded [49]. Although Cu is an essential trace element, its excess induces oxidative stress through the production of reactive oxygen species. Moreover, recent results of a meta-analysis indicate that exposure to high levels of Cu is associated with an increased risk of CVD and coronary heart disease [50]; therefore, a limited consumption of snails by people at risk of or suffering from these diseases is advisable.

As discussed above, Zn concentrations are homogeneous, though not particularly high, satisfying at least 19% of AI in case of snails. Other sources, such as beef meat [11] and oysters, could be considered more interesting to satisfy the nutritional requirements of this essential trace element. In particular, Zn concentration higher than 1000 µg/g wet weight were previously reported in oysters [2].

The percentages of AI for Fe, Zn, and Cu were also calculated for cooked beef sirloin and pork loin, based on the data reported by Lombardi-Boccia et al. [11]. All samples from unconventional animals analyzed in the present study ensure higher percentages of AI with respect to the meat obtained from farm animals, with the exception of Zn.

Due to its toxicity, Pb was included on the list of 10 chemicals of major public health concern by the World Health Organization (WHO) [51]. The most known adverse effects of Pb uptake are developmental neurotoxicity in children and cardiovascular effects and nephrotoxicity in adults [27,52]. In 2010, the EFSA Panel on Contaminants in the Food Chain (CONTAM) claimed that the current provisional tolerable weekly intake (PTWI) of 25 μg/kg of body weight (bw) was no longer appropriate to ensure adequate protection for consumers and concluded that no safe uptake level could be derived; as a consequence, Pb uptake should be as low as possible [53]. Although the majority of the Italian population rarely consumes processed game meat, a restricted population of hunters and their relatives eat wild game meat frequently and could be exposed to elevated Pb intake. In fact, Pb in the H3 sample analyzed in the present study exceeded 17 µg/g. The consumption of 100 g of processed hare meat might determine a Pb intake of 1.73 mg (corresponding to 24 µg/kg bw). On the other hand, using the mean value of 3.39 µg/g or the median value of 0.59 µg/g, a Pb intake of 0.339 mg (corresponding to 4.8 µg/kg bw) or of 0.059 mg (corresponding to 0.84 µg/kg bw) can be obtained, respectively. Only in the latter case, the PTWI of 25 μg/kg bw is not exceeded and, therefore, the negative health effects cannot be excluded in the other two cases.

A considerable amount of information exists regarding the detrimental effects of Cd on human health, and chronic Cd exposure has been associated with kidney disease, osteoporosis, cardiovascular diseases, and cancer [54]. In particular, Cd was ranked seventh on the Priority List of Hazardous Substances by the Agency for Toxic Substances and Disease Registry (ATSDR). A weekly intake of 0.50 μg/kg bw was recently estimated for a representative sample of the Italian population [55]. This value is far below the tolerable weekly intake (TWI) of 2.5 µg/kg bw (175 µg for a reference male of 70 kg, corresponding to a daily intake of 25 µg) established by the EFSA Panel on Contaminants in the Food Chain [55,56]. However, based on the data reported in the present paper, eating 100 g of cooked snails results in a mean daily intake of 33 µg Cd which represents 132% of the aforementioned daily intake, and 100 g of processed woodcock (mean intake of 14 µg Cd) represents 56%. While the consumption of snails can be considered occasional, a monthly consumption of 126 g of woodcock meat has been reported for Italian hunters [9]. On this basis, it can be estimated that Cd intake from the woodcock analyzed in this study can reach a mean value of 211 µg Cd/year in hunters. This represents a small, but not negligible, percentage (3.5%) of the total metal intake as it is higher, for instance, than the intake derived from equine meat which, in Italy, was estimated to be approximately 1% of the total Cd intake [57]. Although Cd from woodcock does not likely constitute a real threat for humans, as it represents only a partial source of food consumed occasionally during the year, environmental Cd contamination, by means of metal accumulation along the food chain, might be envisioned as a risk to human health. To eliminate this source of Cd present in the viscera, it is suggested that woodcock be eviscerated before cooking.

## 4. Materials and Methods

### 4.1. Samples and Cooking Methods

All the samples analyzed were obtained from the Quaderna Valley, a restricted area of the Bologna District (Castel San Pietro-Ozzano Emilia) characterized by low anthropogenic impact, with few exceptions: one pool of snails came from the Treviso District (Silea) and one sample of mushrooms (M1) from the Trento District (Molveno). The anchovy fillets and capers were commercial products. The snails were collected from the wild, while the woodcocks, pheasants, and hares were obtained from local hunters and were shot with Pb-based ammunition. When possible, the shot pellets were removed prior to meal preparation. The pheasants and hares were immediately eviscerated, while the woodcocks were left intact. All the animals were stored frozen at −20 °C. The samples analyzed were prepared using a cooking process following traditional Italian recipes. All the raw samples were manipulated without gloves, washed in tap water, and cooked in steel pans, simulating domestic cooking procedures.

#### 4.1.1. Snails

Before freezing, the wild snails (*Helix pomatia*) were kept at 15–20 °C for 10–12 days to purge the digestive tract. The metal analyses were carried out either on snail samples obtained without any processing (raw meat) or on processed meat. In the first case, five snails were collected from two different sites of the Quaderna Valley and the foot was separated from the viscera while, in the second case, the entire body was used. The snails (five pools of 10 specimens each) were boiled for 20 min, and the soft body was then removed from the shell and cooked again for 30 min in 10 mL of olive oil with 2 g of garlic and 0.5 g of parsley.

#### 4.1.2. Woodcocks

The plucked whole bodies (250–350 g) of the woodcocks (*Scolopax rusticola*; *n* = 5) were roasted separately for two hours in 10 mL of olive oil with 1 g of garlic, 1 g of rosemary and sage. To prepare the woodcock pâtè, the soft part of the cooked meat and the viscera were separated from the bones and blended with 28 g of anchovy fillets and 55 g of capers.

#### 4.1.3. Pheasants

The eviscerated and plucked pheasants (800–1000 g; *Phasianus colchicus; n* = 8) were roasted separately as previously described for 10 min; 50 g of minced carrot, 50 g of minced onion, 20 g of minced celery, and 150 mL of white wine were then added to the preparation, and it was cooked for two hours. Sample P4 was also seasoned with 200 g of mushrooms obtained from sample M1.

#### 4.1.4. Hares

The eviscerated and skinned hares (2–2.8 kg; *Lepus europaeus*; *n* = 6) were seasoned separately with one liter of red wine, 100 g of minced carrots, 100 g of minced onions, and 10 g of garlic, and left to marinate for 36 h at 4 °C. The meat was then roasted as described above for 10 min; the marinade was added to the meat and the preparation was cooked for an additional two hours.

#### 4.1.5. Mushrooms

Fresh mushrooms (*n* = 7) from a mixture of 50% *Macrolepiota procera* and 50% *Boletus edulis* (M1), *Cyclocybe aegerita* (M2 and M6), *Boletus edulis* (M3), *Pleurotus ostreatus* (M4), *Armillaria mellea* (M5), and *Cantharellus cibarius* (M7) were cooked for 30 min in 10 mL of olive oil with 2 g of garlic and 0.5 g of parsley.

### 4.2. Ethics Statement

Licensed hunters shot the game animals analyzed in this study during the established hunting season and in accordance with Italian law. Ethical approval is not required for research with *H. pomatia*. This study did not involve endangered or protected species.

### 4.3. Trace Element Analysis

The samples were digested using a wet procedure in polypropylene tubes (DigiTUBES SCP Science, Baie-D’Urfe, QC, Canada). Ten mL of 70% nitric acid (J.T. Baker Instra-Analyzed™ Phillipsburg, NJ, USA) were added, and the tubes were placed in Digi-Prep graphite Digestion Blocks (SCP Science, Baie-D’Urfe, QC, Canada) at 75 °C overnight. After cooling, the clear solutions were diluted to 20 mL with high purity deionized water (Evoqua Water Technologies, Phillipsburg, United States). The samples were additionally diluted with a solution of 2% nitric acid and 0.5% hydrochloric acid (Sigma, Suprapur^®,^ St. Louis, MO, United States). The analyses were carried out using inductively coupled plasma mass spectrometry (ICP-MS 7700 Series Agilent Technologies Inc, Santa Clara, CA, USA) with an ASX-500 CETAC Autosampler (Cetac Technologies, Omaha, NE, USA). The operating parameters included an Rf power of 1.55 kW, a plasma (Ar) gas flow of 15 L/min, a carrier gas flow (Ar) of 1.01 mL/min, a cell gas flow (He) for “He” mode of 5 mL/min and a gas flow for “HeHe” mode of 10 mL/min. In the “No gas mode”, no He flow passed through the cell. The isotopes (*m/z*) monitored in the “No gas mode” were 27Al, 60Ni, 63Cu, and 111Cd; the isotopes (*m/z*) monitored in the “He mode” were 55Mn, 59Co, 66Zn, 78Se, 98Mo, 202Hg, and 208Pb, while 52Cr, 56Fe, and 75As were monitored in the “HeHe” mode. The concentrations were calculated using solvent calibration curves. The calibration standards were from Agilent Technologies Inc. (Santa Clara, CA, USA). A mixture of internal standards (each one having a concentration of 1 µg/mL) was infused continuously by an alternative means of entrance using ICP-MS (code 5183-4681 Agilent Technologies Inc Santa Clara, CA, USA) to quantify the samples.

The accuracy of the method was determined by analyzing the certified reference material (Joint Research Centre BCR-185R Bovine Liver) in each batch. The concentration values of the reference materials fell within the confidence interval given by the Joint Research Centre (Brussels, Belgium). For each series of analyses, a white sample (acid used for sample mineralization) was mineralized and treated as described above; the limit of quantification (LOQ) was 0.005 µg/g, and the limit of detection (LOD) was 0.003 µg/g for each element analyzed. The results were expressed in µg/g wet weight (ww).

### 4.4. Statistical Analysis

The mean, median, standard deviation, and minimum and maximum values were calculated for the descriptive statistics. Values below the limit of quantification (0.05 µg/g) were set at 0.5 LOQ to carry out the statistical analyses on all the data available. The Kruskal–Wallis rank sum test was applied to compare the concentration of each element among all the groups of raw and processed samples (snails, woodcocks, hare, pheasant); adjusted *p*-values were calculated for multiple comparison, and values lower than 0.05 were considered statistically significant. Spearman correlation coefficients (r) among the elements were also reported with their *p*-values. As regards the benefit-risk calculations, the exposure to the non-essential trace elements Pb and Cd from the dietary source of a 70 kg male (assuming 100 g of the specific processed and cooked meat) was simulated.

## 5. Conclusions

Discussion regarding the benefits and the risks associated with the consumption of meat from unconventional animals is still in its infancy. The increasing demand for food will prompt both producers and consumers to seek alternative food sources of high nutritional value; in particular, snails could be considered as a good candidate with, however, avoiding the viscera, which contain high concentrations of non-essential trace elements, such as Al, Cr, Cd, and Pb. Moreover, non-lead ammunition is recommended to ensure not only the health protection of the consumer but also that of the terrestrial and aquatic fauna.

The analyses of metal concentrations in home-processed food deserve more attention, since the use of particular seasonings in preparing certain dishes can add an unexpected metal burden, as evidenced in the case of woodcock pâtè. Therefore, one must be careful in reaching conclusions regarding the dietary metal intake of a food, beginning with the data obtained from raw sources.

## Figures and Tables

**Table 1:** Concentrations of essential and non-essential trace elements in five samples (S1–S5) of processed snails. Each sample was obtained by pooling 10 snails. Data are reported as µg/g wet weight. LOQ, limit of quantification; SD, standard deviation.

	Essential Trace Elements								Non-Essential Trace Elements					
	Fe	Zn	Cu	Mn	Se	Ni	Mo	Co	Pb	Cd	Hg	Al	As	Cr
S1	81.35	34.90	87.35	32.97	0.144	0.127	0.345	0.071	0.329	0.404	<LOQ	8.770	0.010	0.103
S2	56.45	21.64	134.4	8.000	0.114	0.205	0.250	0.050	0.112	0.460	<LOQ	22.70	0.030	0.216
S3	37.14	20.63	53.67	21.10	0.087	0.187	0.380	0.070	0.181	0.209	<LOQ	4.800	0.020	0.318
S4	230.9	18.06	18.25	41.20	0.085	0.676	0.330	0.173	0.253	0.180	<LOQ	126.3	0.046	0.748
S5	92.35	24.28	82.00	24.20	0.104	0.296	0.350	0.101	0.198	0.375	<LOQ	43.00	0.030	0.311
Median	81.35	21.64	82.00	24.20	0.104	0.205	0.345	0.071	0.198	0.375	<LOQ	47.58	0.030	0.311
Mean	99.64	23.90	75.13	25.49	0.107	0.298	0.331	0.093	0.215	0.326	<LOQ	41.11	0.027	0.339
SD	76.47	6.539	43.03	12.55	0.024	0.220	0.049	0.048	0.081	0.124	<LOQ	52.56	0.013	0.245

**Table 2:** Concentrations of essential and non-essential trace elements in five samples (W1–W5) of processed woodcocks. Data are reported as µg/g wet weight.

	Essential Trace Elements								Non-Essential Trace Elements					
	Fe	Zn	Cu	Mn	Se	Ni	Mo	Co	Pb	Cd	Hg	Al	As	Cr
W1	48.25	8.960	1.600	0.974	0.465	0.140	0.034	0.022	0.793	0.078	0.049	4.000	0.445	0.048
W2	104.4	9.560	1.770	1.470	0.447	0.145	0.045	0.058	0.589	0.091	0.069	54.00	0.257	0.145
W3	55.79	10.10	1.640	1.490	0.650	0.070	0.099	0.026	2.421	0.066	0.043	16.72	0.371	0.062
W4	46.20	11.30	2.000	1.100	0.536	0.075	0.053	0.026	0.407	0.221	0.059	2.300	0.790	0.049
W5	62.80	12.30	2.000	1.200	0.818	0.429	0.085	0.031	0.505	0.247	0.053	5.900	0.779	0.209
Median	55.79	10.10	1.770	1.200	0.536	0.140	0.053	0.026	0.589	0.091	0.053	5.900	0.445	0.062
Mean	63.49	10.44	1.802	1.247	0.583	0.172	0.063	0.033	0.943	0.141	0.055	16.58	0.528	0.103
SD	23.80	1.349	0.191	0.228	0.154	0.148	0.028	0.015	0.838	0.086	0.010	21.65	0.243	0.072

**Table 3:** Concentrations of essential and non-essential trace elements in eight samples (P1–P8) of processed pheasants. Data are reported as µg/g wet weight.

	Essential Trace Elements								Non-Essential Trace Elements					
	Fe	Zn	Cu	Mn	Se	Ni	Mo	Co	Pb	Cd	Hg	Al	As	Cr
P1	13.39	8.680	0.774	0.644	0.120	0.046	0.032	0.011	0.010	<LOQ	<LOQ	0.584	0.007	0.046
P2	28.68	12.12	3.490	1.210	0.090	0.209	0.060	0.012	0.092	0.028	<LOQ	2.900	0.090	0.057
P3	13.18	16.60	1.120	0.511	0.358	0.072	0.351	0.009	0.310	0.013	<LOQ	2.100	<LOQ	0.036
P4	28.40	11.00	2.500	1.200	0.836	0.180	0.045	0.010	0.163	0.083	0.179	2.900	0.028	0.108
P5	17.47	9.020	0.720	0.820	0.259	0.056	0.310	0.005	0.030	<LOQ	<LOQ	0.928	<LOQ	0.032
P6	6.010	8.294	0.688	0.087	0.252	<LOQ	0.034	0.006	0.010	<LOQ	<LOQ	0.009	<LOQ	0.016
P7	15.73	9.640	0.891	0.581	0.220	0.341	0.047	0.014	0.013	<LOQ	<LOQ	0.388	0.005	0.067
P8	27.23	12.38	0.800	0.374	0.172	0.011	0.030	<LOQ	0.470	0.010	<LOQ	1.900	<LOQ	0.032
Median	16.60	10.32	0.846	0.613	0.236	0.072	0.046	0.010	0.061	0.021	<LOQ	1.414	0.018	0.041
Mean	18.76	10.97	1.373	0.678	0.288	0.131	0.114	0.010	0.137	0.034	<LOQ	1.464	0.033	0.049
SD	8.422	2.799	1.097	0.419	0.245	0.122	0.141	0.003	0.175	0.034	<LOQ	1.167	0.044	0.031

**Table 4:** Concentrations of essential and non-essential trace elements in six samples (H1–H6) of processed hares. Data are reported as µg/g wet weight.

	Essential Trace Elements								Non-Essential Trace Elements					
	Fe	Zn	Cu	Mn	Se	Ni	Mo	Co	Pb	Cd	Hg	Al	As	Cr
H1	29.47	32.17	3.000	0.434	0.196	0.009	0.033	0.005	0.019	<LOQ	<LOQ	0.595	<LOQ	0.015
H2	50.30	23.40	3.000	0.786	0.485	0.049	0.033	0.009	1.800	0.006	<LOQ	0.950	0.013	0.049
H3	28.00	10.70	2.200	0.755	0.234	0.149	0.057	0.008	17.30	0.022	0.006	1.600	0.075	0.065
H4	16.23	8.270	1.190	1.270	0.065	0.070	0.035	0.005	0.057	<LOQ	0.010	3.740	<LOQ	0.043
H5	57.29	11.57	3.180	1.200	0.210	0.168	0.105	0.011	1.110	0.006	0.060	9.870	0.016	0.117
H6	21.16	9.60	1.650	0.910	0.100	0.097	0.027	0.005	0.083	<LOQ	<LOQ	2.250	0.013	0.057
Median	28.74	11.14	2.600	0.848	0.203	0.084	0.034	0.007	0.597	0.006	0.010	1.925	0.015	0.053
Mean	33.74	15.95	2.370	0.893	0.215	0.090	0.048	0.007	3.395	0.011	0.025	3.168	0.029	0.058
SD	16.40	9.640	0.823	0.309	0.148	0.060	0.030	0.003	6.850	0.009	0.030	3.467	0.030	0.034

**Table 5:** Concentrations of essential and non-essential trace elements in three samples of anchovies (A1–A3) and three samples of capers (C1–C3) used for the preparation of woodcock pâté. Data are reported as µg/g wet weight.

	Essential Trace Elements								Non-Essential Trace Elements					
	Fe	Zn	Cu	Mn	Se	Ni	Mo	Co	Pb	Cd	Hg	Al	As	Cr
A1	18.99	24.55	1.970	1.150	0.530	0.029	0.011	0.017	0.048	0.023	0.143	<LOQ	1.880	0.027
A2	18.66	19.36	1.420	0.975	0.583	0.031	0.011	0.017	0.022	0.027	0.121	0.232	4.500	0.024
A3	15.35	18.71	1.940	0.677	0.545	0.025	0.009	0.014	0.034	0.019	0.142	0.361	1.700	0.021
Median	18.66	19.36	1.940	0.975	0.545	0.029	0.011	0.016	0.034	0.023	0.142	0.297	1.880	0.024
Mean	17.66	20.87	1.777	0.934	0.553	0.028	0.010	0.016	0.035	0.023	0.135	0.297	2.693	0.024
SD	2.013	3.201	0.309	0.239	0.027	0.003	0.001	0.002	0.013	0.004	0.012	0.091	1.567	0.003
C1	6.770	1.190	1.310	0.670	0.048	0.123	0.071	0.031	0.072	<LOQ	<LOQ	1.620	<LOQ	0.090
C2	6.740	0.969	0.980	0.530	0.112	0.110	0.040	0.030	0.077	<LOQ	<LOQ	1.700	<LOQ	0.050
C3	6.800	0.930	1.330	0.260	0.089	0.080	0.059	0.031	0.035	<LOQ	<LOQ	1.800	<LOQ	0.052
Median	6.770	0.969	1.310	0.530	0.089	0.110	0.059	0.031	0.072	<LOQ	<LOQ	1.700	<LOQ	0.052
Mean	6.770	1.030	1.207	0.487	0.083	0.104	0.057	0.031	0.061	<LOQ	<LOQ	1.707	<LOQ	0.064
SD	0.030	0.140	0.197	0.208	0.032	0.022	0.016	0.000	0.023	<LOQ	<LOQ	0.090	<LOQ	0.023

**Table 6:** Concentrations of essential and non-essential trace elements in seven samples (M1–M7) of mushrooms used as seasonings. Data are reported as µg/g wet weight.

	Essential Trace Elements								Non-Essential Trace Elements					
	Fe	Zn	Cu	Mn	Se	Ni	Mo	Co	Pb	Cd	Hg	Al	As	Cr
M1	14.80	12.39	5.300	2.660	0.855	0.171	0.037	0.018	0.094	0.197	0.522	5.330	0.041	0.101
M2	3.400	10.13	1.700	0.544	0.015	0.011	0.016	<LOQ	0.011	0.132	0.010	1.400	<LOQ	0.025
M3	21.03	10.49	4.640	1.200	0.116	0.093	0.062	0.013	0.107	0.086	0.028	14.07	0.018	0.126
M4	17.52	12.15	0.690	1.020	0.071	0.030	<LOQ	0.006	0.014	0.082	0.033	6.230	0.014	0.034
M5	9.100	5.250	1.390	1.000	0.035	0.031	0.035	0.010	0.031	0.213	0.006	1.240	<LOQ	0.035
M6	7.940	12.67	3.530	0.650	0.014	0.044	0.040	<LOQ	0.013	0.119	0.013	5.040	<LOQ	0.020
M7	23.59	15.89	6.300	0.670	0.018	0.640	0.034	0.012	0.104	0.039	<LOQ	16.52	0.034	0.171
Median	14.80	12.15	3.530	1.000	0.035	0.044	0.036	0.012	0.031	0.119	0.021	5.330	0.026	0.035
Mean	13.91	11.28	3.364	1.106	0.161	0.146	0.037	0.012	0.053	0.124	0.102	7.119	0.027	0.073
SD	7.388	3.253	2.154	0.725	0.308	0.225	0.015	0.004	0.046	0.063	0.206	5.948	0.013	0.060

**Table 7:** Percentages of the recommended adequate intakes (AI) calculated for Fe, Zn, Cu, Mn, Se, and Co. The data were obtained considering the consumption of 100 g of the processed meat.

	Fe *		Zn	Cu *		Mn	Se	Co
**Snail**	90	*62*	19	468	*577*	83	15	51
**Woodcock**	57	*39*	8	11	*13*	4	83	10
**Pheasant**	17	*12*	9	9	*11*	2	41	17
**Hare**	30	*21*	13	15	*18*	3	31	7
**Beef#**	28	*19*	44	5	*6*	-	-	-
**Pork#**	6		20	4	*5*	-	-	-

Percentages were calculated according to the adequate intakes (AI) reported by EFSA (European Food Safety Authority) [10]. The following AI were used as reference: Fe, 11 mg/d for man and post-menopausal women and 16 for pre-menopausal women; Zn, 12.7 mg/d; Cu 1.6 mg/d for man and 1.3 for women; Mn, 3 mg/d; Se, 70 µg/d and Co, 65 µg/d. * The AI for Fe and Cu reported in italics are referred to women. # The AI for cooked beef and pork were calculated from the concentrations of Fe, Zn, and Cu reported for beef sirloin and pork loin by Lombardi-Boccia et al. [11].

## Supplementary Materials

Supplementary materials can be found at https://www.mdpi.com/2075-1729/10/5/75/s1. The concentrations of essential and non-essential trace elements in foot and viscera of raw snails are reported in Table S1. The concentrations of essential and non-essential trace elements in the seasonings used for the traditional recipes are reported in Table S2., The correlations among the essential and non-essential trace elements analysed in processed meat from unconventional animals are reported in Table S3.

## Abbreviations

AI	Adequate Intake
ATSDR	Agency for Toxic Substances and Disease Registry
bw	Body Weight
CVDs	Cardiovascular Diseases
EFSA	European Food Safety Authority
ICP-MS	Inductively Coupled Plasma Mass Spectrometry
LOD	Limit of Detection
LOQ	Limit of Quantification
MeHg	Methylmercury
ML	Maximum Admissible Level
MnSOD	Manganese Superoxide Dismutase
PRI	Population Reference Intake
PTWI	Provisional Tolerable Weekly Intake
ROS	Reactive Oxygen Species
WHO	World Health Organization

## References

[1] Mwangi M.N., Oonincx D.G.A.B., Stouten T., Veenenbos M., Melse-Boonstra A., Dicke M., Van Loon J.J.A. (2018). Insects as sources of iron and zinc in human nutrition. Nutr. Res. Rev..

[2] Carpenè E., Andreani G., Isani G. (2017). Trace elements in unconventional animals: A 40-year experience. J. Trace Elements Med. Biol..

[3] Bastias-Montes J.-M., Balladares P., Acuña-Nelson S.-M., Quevedo R., Munoz O. (2017). Determining the effect of different cooking methods on the nutritional composition of salmon (*Salmo salar*) and chilean jack mackerel (*Trachurus murphyi*) fillets. PLoS ONE.

[4] Ribeiro D.M., Mourato M.P., Almeida A.M. (2019). Assessing mineral status in edible tissues of domestic and game animals: A review with a special emphasis in tropical regions. Trop. Anim. Heal. Prod..

[5] Cawthorn N.-M., Hoffman L.C. (2016). Controversial cuisine: A global account of the demand, supply and acceptance of “unconventional” and “exotic” meats. Meat Sci..

[6] Zoroddu M.A., Aaseth J., Crisponi G., Medici S., Peana M., Nurchi V.M. (2019). The essential metals for humans: A brief overview. J. Inorg. Biochem..

[7] World Health Organization (1996). Trace Elements in Human Nutrition and Health.

[8] Alves R.N., Maulvault A.L., Barbosa V., Fernández-Tejedor M., Tediosi A., Kotterman M., Heuvel F.H.V.D., Robbens J., Fernandes J.O., Rasmussen R.R. (2018). Oral bioaccessibility of toxic and essential elements in raw and cooked commercial seafood species available in European markets. Food Chem..

[9] Ferri M., Baldi L., Cavallo S., Pellicanò R., Brambilla G. (2017). Wild game consumption habits among Italian shooters: Relevance for intakes of cadmium, perfluorooctanesulfonic acid, and 137 cesium as priority contaminants. Food Addit. Contam. Part A.

[10] European Food Safety Authority (EFSA) (2017). Dietary reference values for nutrients. Summary report. EFSA Support. Publ..

[11] Lombardi-Boccia G., Lanzi S., Aguzzi A. (2005). Aspects of meat quality: Trace elements and B vitamins in raw and cooked meats. J. Food Compos. Anal..

[12] Laird B., Chan H.-M. (2013). Bioaccessibility of metals in fish, shellfish, wild game, and seaweed harvested in British Columbia, Canada. Food Chem. Toxicol..

[13] Ćirić J., Cerić O., Marković R., Janjić J., Spirić D., Popović M., Pećanac B., Baltić B., Baltić M.Ž. (2018). Seasonal distributions of heavy metal concentrations in different snail (*Helix pomatia*) tissues from an urban environment in Serbia. Environ. Sci. Pollut. Res..

[14] Nica D., Draghici G.A., Andrica F., Popescu S., Coricovac D.E., Dehelean C., Gergen I.I., Kovatsi L., Coleman M.D., Tsatsakis A.M. (2019). Short-term effects of very low dose cadmium feeding on copper, manganese and iron homeostasis: A gastropod perspective. Environ. Toxicol. Pharmacol..

[15] Ogbuagu M. (2011). The nutrient composition of flesh of giant african land snail: *Achantina achantina*. Electron. J. Environ. Agric. Food Chem..

[16] Höckner M., Stefanon K., De Vaufleury A., Monteiro F., Pérez-Rafael S., Palacios Ò., Capdevila M., Atrian S., Dallinger R. (2011). Physiological relevance and contribution to metal balance of specific and non-specific Metallothionein isoforms in the garden snail, *Cantareus aspersus*. BioMetals.

[17] Salvato B., Zatta P., Ghiretti-Magaldi A., Ghiretti F. (1973). On the active site of hemocyanin. FEBS Lett..

[18] Dewilde S., Winnepenninckx B., Arndt M.H.L., Nascimento D.G., Santoro M.M., Knight M., Miller A.N., Kerlavage A.R., Geoghagen N., Van Marck E. (1998). Characterization of the Myoglobin and Its Coding Gene of the Mollusc *Biomphalaria glabrata*. J. Biol. Chem..

[19] Soldati A.L., Jacob D., Glatzel P., Swarbrick J.C., Geck J. (2016). Element substitution by living organisms: The case of manganese in mollusc shell aragonite. Sci. Rep..

[20] Carpenè E., Serra R., Isani G. (1995). Heavy metals in some species of waterfowl of Northern Italy. J. Wildl. Dis..

[21] Dalla Libera L., Carpenè E. (1997). Myosin heavy and light chains and myosin light chain kinase in skeletal and smooth muscle of some wild avian species. Comp. Biochem. Physiol. Part B Biochem. Mol. Biol..

[22] Holley A.K., Bakthavatchalu V., Velez-Roman J.M., Clair D.K.S. (2011). Manganese superoxide dismutase: guardian of the powerhouse. Int. J. Mol. Sci..

[23] Vallee B.L., Falchuk K.H. (1993). The biochemical basis of zinc physiology. Physiol. Rev..

[24] Maret W. (2017). Zinc in cellular regulation: the nature and significance of “zinc signals”. Int. J. Mol. Sci..

[25] Vašák M., Meloni G. (2011). Chemistry and biology of mammalian metallothioneins. JBIC J. Boil. Inorg. Chem..

[26] Ziomek M., Drozd L., Chałabis-Mazurek A., Szkucik K., Paszkiewicz W., Valverde Piedra J.L., Bełkot Z., Maćkowiak-Dryka M., Gondek M., Knysz P. (2018). Concentration levels of cadmium and lead in the raw and processed meat of *Helix pomatia* snails. Pol. J. Vet. Sci..

[27] Green R.E., Pain D.J. (2019). Risks to human health from ammunition-derived lead in Europe. Ambio.

[28] Andreotti A., Borghesi F., Aradis A. (2016). Lead ammunition residues in the meat of hunted woodcock: A potential health risk to consumers. Ital. J. Anim. Sci..

[29] Gerofke A., Ulbig E., Martin A., Müller-Graf C., Selhorst T., Gremse C., Spolders M., Schafft H., Heinemeyer G., Greiner M. (2018). Lead content in wild game shot with lead or non-lead ammunition—Does “state of the art consumer health protection” require non-lead ammunition?. PLoS ONE.

[30] Danieli P.P., Serrani F., Primi R., Ponzetta M.P., Ronchi B., Amici A. (2012). Cadmium, Lead, and Chromium in large game: a local-scale exposure assessment for hunters consuming meat and liver of wild boar. Arch. Environ. Contam. Toxicol..

[31] Roselli C., Desideri D., Meli M.A., Fagiolino I., Feduzi L. (2016). Essential and toxic elements in meat of wild birds. J. Toxicol. Environ. Heal. Part A.

[32] Ertl K., Kitzer R., Goessler W. (2016). Elemental composition of game meat from Austria. Food Addit. Contam. Part B.

[33] Regulation EC No 1881/2006 Setting Maximum Levels for Certain Contaminants in Food Stuffs. https://eur-lex.europa.eu/legal-content/EN/ALL/?uri=celex:32006R1881.

[34] Pain D.J., Cromie R.L., Newth J., Brown M.J., Crutcher E., Hardman P., Hurst L., Mateo R., Meharg A.A., Moran A.C. (2010). Potential hazard to human health from exposure to fragments of lead bullets and shot in the tissues of game animals. PLoS ONE.

[35] González-Weller D., Karlsson L., Caballero A., Hernandez F., Gutiérrez A.J., González-Iglesias T., Marino M., Hardisson A. (2006). Lead and cadmium in meat and meat products consumed by the population in Tenerife Island, Spain. Food Addit. Contam..

[36] Cirillo T., Cocchieri R.A., Fasano E., Lucisano A., Tafuri S., Ferrante M.C., Carpenè E., Andreani G., Isani G. (2011). Cadmium accumulation and antioxidant responses in *Sparus aurata* exposed to waterborne cadmium. Arch. Environ. Contam. Toxicol..

[37] Isani G., Carpenè E. (2014). Metallothioneins, unconventional proteins from unconventional animals: a long journey from nematodes to mammals. Biomolecules.

[38] Orlando P., Silvestri S., Ferlizza E., Andreani G., Carpene E., Falcioni G., Tiano L., Isani G. (2017). Biochemical responses to cadmium exposure in *Oncorhynchus mykiss* erythrocytes. Ecotoxicol. Environ. Saf..

[39] Carpene E., Andreani G., Monari M., Castellani G., Isani G. (2006). Distribution of Cd, Zn, Cu and Fe among selected tissues of the earthworm (*Allolobophora caliginosa*) and Eurasian woodcock (*Scolopax rusticola*). Sci. Total. Environ..

[40] (2012). European Food Safety Authority (EFSA) Scientific opinion on the risk for public health related to the presence of mercury and methylmercury in food. EFSA J..

[41] McLean E., Cogswell M., Egli I., Wojdyla D., De Benoist B. (2008). Worldwide prevalence of anaemia, WHO Vitamin and Mineral Nutrition Information System, 1993–2005. Public Heal. Nutr..

[42] Beck K.L., Conlon C.A., Kruger R., Heath A.-L.M. (2014). Dietary Determinants of and Possible Solutions to Iron Deficiency for Young Women Living in Industrialized Countries: A Review. Nutrients.

[43] Fleming D.J., Jacques P.F., E Dallal G., Tucker K.L., Wilson P.W., Wood R.J. (1998). Dietary determinants of iron stores in a free-living elderly population: The Framingham Heart Study. Am. J. Clin. Nutr..

[44] Czerwonka M., Tokarz A. (2017). Iron in red meat–friend or foe. Meat Sci..

[45] European Food Safety Authority (EFSA) (2015). Scientific opinion on dietary reference values for iron. EFSA J..

[46] Rohrmann S., Linseisen J. (2016). Processed meat: The real villain?. Proc. Nutr. Soc..

[47] Pacheco D.A.Q., Sookthai D., Wittenbecher C., Graf M.E., Schübel R., Johnson T., Katzke V., Jakszyn P., Kaaks R., Kuhn T. (2018). Red meat consumption and risk of cardiovascular diseases—Is increased iron load a possible link?. Am. J. Clin. Nutr..

[48] European Food Safety Authority (EFSA) (2015). Scientific opinion on dietary reference values for copper. EFSA J..

[49] European Food Safety Authority (EFSA) (2006). Tolerable Upper Intake Level on Vitamins and Minerals.

[50] Chowdhury R., Ramond A., O’Keeffe L.M., Shahzad S., Kunutsor S.K., Muka T., Gregson J., Willeit P., Warnakula S., Khan H. (2018). Environmental toxic metal contaminants and risk of cardiovascular disease: systematic review and meta-analysis. BMJ.

[51] WHO Ten Chemicals of Major Public Health Concern. http://www.who.int/ipcs/features/10chemicals_en.pdf?ua=1.

[52] Gidlow D. (2015). Lead toxicity. Occup. Med..

[53] European Food Safety Authority (EFSA) (2010). Scientific opinion on lead in food. EFSA J..

[54] Jacobo-Estrada T.L., Santoyo-Sanchez M.P., Thévenod F., Barbier O. (2017). Cadmium handling, toxicity and molecular targets involved during pregnancy: lessons from experimental models. Int. J. Mol. Sci..

[55] Filippini T., Cilloni S., Malavolti M., Violi F., Malagoli C., Tesauro M., Bottecchi I., Ferrari A., Vescovi L., Vinceti M. (2018). Dietary intake of cadmium, chromium, copper, manganese, selenium and zinc in a Northern Italy community. J. Trace Elements Med. Biol..

[56] European Food Safety Authority (EFSA) (2012). Cadmium dietary exposure in the European population. EFSA J..

[57] Baldini M., Stacchini P., Cubadda F., Miniero R., Parodi P., Facelli P. (2000). Cadmium in organs and tissues of horses slaughtered in Italy. Food Addit. Contam..

