# Supplementary material for: Trace Elements in Home-Processed Food Obtained from Unconventional Animals
Source: Life (Basel). 2020 May 23;10(5):75. doi: 10.3390/life10050075 (PMC7281459; doi:10.3390/life10050075)
Supplement: Supplementary file 1 [file life-10-00075-s001.zip › life-762977-supplementary.docx]

**Supplmantary Materials of Trace Elements in Home-Processed Food Obtained from Unconventional Animals**

**Table S1.** Essential and non-essential trace elements concentrations in foot (F) and viscera (V) of raw snails sampled in two different sites (1 and 2) of the Quaderna Valley. For each site, five snails were collected. Data are reported in µg/g wet weight.

|  |  | Essential Trace Elements | | | | | | | | Non-Essential Trace Elements | | | | | |
| --- | --- | --- | --- | --- | --- | --- | --- | --- | --- | --- | --- | --- | --- | --- | --- |
|  |  | Fe | Zn | Cu | Mn | Se | Ni | Mo | Co | Pb | Cd | Hg | Al | As | Cr |
| **F1** | Mean | 28.55 | 8.113 | 30.05 | 0.963 | 0.032 | 0.041 | 0.041 | 0.010 | 0.005 | 0.051 | <LOQ | 2.370 | <LOQ | 0.024 |
|  | Median | 18.14 | 8.180 | 13.77 | 1.040 | 0.032 | 0.045 | 0.037 | 0.009 | 0.005 | 0.009 | <LOQ | 2.340 | <LOQ | 0.020 |
|  | SD | 19.18 | 1.451 | 28.91 | 0.150 | 0.019 | 0.007 | 0.008 | 0.002 | <LOQ | 0.075 | <LOQ | 1.235 | <LOQ | 0.011 |
|  | Min | 16.82 | 6.630 | 12.94 | 0.790 | 0.013 | 0.033 | 0.036 | 0.009 | 0.005 | 0.006 | <LOQ | 1.150 | <LOQ | 0.015 |
|  | Max | 50.68 | 9.530 | 63.43 | 1.060 | 0.050 | 0.046 | 0.051 | 0.013 | 0.005 | 0.137 | <LOQ | 3.620 | <LOQ | 0.036 |
| **F2** | Mean | 14.22 | 8.610 | 24.18 | 1.207 | 0.043 | 0.025 | 0.073 | 0.008 | 0.029 | 0.030 | <LOQ | 1.088 | 0.007 | 0.015 |
|  | Median | 15.38 | 8.660 | 20.79 | 0.679 | 0.042 | 0.027 | 0.078 | 0.008 | 0.029 | 0.030 | <LOQ | 0.432 | 0.007 | 0.016 |
|  | SD | 3.221 | 0.114 | 13.83 | 0.973 | 0.011 | 0.007 | 0.021 | 0.000 | 0.022 | 0.008 | <LOQ | 1.340 | 0.002 | 0.005 |
|  | Min | 10.58 | 8.480 | 12.35 | 0.611 | 0.033 | 0.017 | 0.050 | 0.008 | 0.007 | 0.024 | <LOQ | 0.203 | 0.005 | 0.009 |
|  | Max | 16.70 | 8.690 | 39.39 | 2.330 | 0.055 | 0.031 | 0.091 | 0.008 | 0.050 | 0.035 | <LOQ | 2.630 | 0.008 | 0.019 |
| **V1** | Mean | 94.17 | 24.02 | 31.41 | 12.77 | 0.083 | 0.128 | 0.290 | 0.063 | 0.092 | 1.288 | <LOQ | 39.53 | 0.012 | 0.089 |
|  | Median | 68.43 | 21.42 | 20.03 | 12.18 | 0.089 | 0.082 | 0.310 | 0.049 | 0.088 | 0.562 | <LOQ | 19.55 | 0.006 | 0.049 |
|  | SD | 72.60 | 10.63 | 27.18 | 3.261 | 0.024 | 0.081 | 0.039 | 0.031 | 0.013 | 1.514 | <LOQ | 43.57 | 0.011 | 0.070 |
|  | Min | 38.92 | 14.93 | 11.77 | 9.850 | 0.057 | 0.081 | 0.245 | 0.042 | 0.082 | 0.274 | <LOQ | 9.530 | 0.005 | 0.047 |
|  | Max | 176.8 | 35.70 | 62.44 | 16.29 | 0.104 | 0.222 | 0.316 | 0.099 | 0.107 | 3.029 | <LOQ | 89.50 | 0.025 | 0.170 |
| **V2** | Mean | 334.6 | 69.35 | 24.28 | 48.50 | 0.119 | 0.823 | 0.587 | 0.260 | 0.591 | 0.575 | <LOQ | 18.44 | 0.108 | 0.761 |
|  | Median | 115.1 | 70.41 | 24.91 | 44.50 | 0.123 | 0.382 | 0.525 | 0.207 | 0.600 | 0.636 | <LOQ | 18.64 | 0.020 | 0.137 |
|  | SD | 402.3 | 56.89 | 7.256 | 15.54 | 0.016 | 0.875 | 0.165 | 0.163 | 0.146 | 0.368 | <LOQ | 7.156 | 0.153 | 1.081 |
|  | Min | 89.80 | 11.93 | 16.73 | 35.35 | 0.101 | 0.256 | 0.462 | 0.130 | 0.441 | 0.180 | <LOQ | 13.58 | 0.019 | 0.037 |
|  | Max | 798.9 | 125.7 | 38.20 | 65.65 | 0.133 | 1.830 | 0.774 | 0.443 | 0.732 | 0.909 | <LOQ | 23.76 | 0.284 | 2.010 |

**Table S2.** Essential and non-essential trace elements concentrations in the most commons seasonings used for the traditional recipes. Data are reported in µg/g wet weight.

|  | Essential Trace Elements | | | | | | | | Non-Essential Trace Elements | | | | | |
| --- | --- | --- | --- | --- | --- | --- | --- | --- | --- | --- | --- | --- | --- | --- |
|  | Fe | Zn | Cu | Mn | Se | Ni | Mo | Co | Pb | Cd | Hg | Al | As | Cr |
| Garlic | 7.890 | 8.680 | 2.440 | 2.350 | 0.007 | 0.050 | 0.440 | <LOQ | 0.012 | <LOQ | <LOQ | <LOQ | 0.008 | 0.031 |
| Onion | 3.240 | 2.380 | 1.027 | 1.150 | <LOQ | 0.042 | 0.300 | <LOQ | 0.018 | <LOQ | <LOQ | <LOQ | <LOQ | 0.081 |
| Carrot | 5.120 | 2.760 | 1.070 | 1.540 | <LOQ | 0.031 | 0.120 | <LOQ | 0.026 | <LOQ | <LOQ | 0.649 | <LOQ | 0.115 |
| Celery | 41.64 | 4.820 | 1.350 | 6.700 | 0.008 | 0.150 | 2.590 | 0.016 | 0.078 | 0.010 | <LOQ | 21.75 | 0.024 | 0.159 |
| Rosemary | 9.520 | 3.730 | 1.490 | 2.960 | 0.007 | 0.067 | 0.070 | <LOQ | 0.020 | <LOQ | <LOQ | 3.450 | 0.006 | 0.048 |
| Sage | 52.33 | 5.980 | 1.680 | 8.700 | <LOQ | 0.122 | 0.980 | 0.021 | 0.072 | <LOQ | <LOQ | 23.80 | 0.030 | 0.187 |

**Table S3**. Correlations among the essential and non-essential trace elements analysed in cooked food from unconventional animals. Values above the diagonal correspond to the *p* values, while the values below the diagonal correspond to the coefficients of correlation (r).

|  | **Fe** | **Zn** | **Cu** | **Mn** | **Ni** | **Mo** | **Se** | **Co** | **Pb** | **Cd** | **Hg** | **Al** | **As** | **Cr** |
| --- | --- | --- | --- | --- | --- | --- | --- | --- | --- | --- | --- | --- | --- | --- |
| **Fe** |  | 0.009 | 6.21 × 10^−5^ | 2.87 × 10^−5^ | 0.010 | 0.061 | 0.863 | 3.15 × 10^−5^ | 0.004 | 3.1× 10^−5^ | 0.161 | 3.40 × 10^−7^ | 0.001 | 0.001 |
| **Zn** | 0.52 |  | 1.48E−05 | 0.144 | 0.211 | 0.032 | 0.472 | 0.073 | 0.268 | 0.007 | 0.100 | 0.146 | 0.826 | 0.080 |
| **Cu** | 0.72 | 0.76 |  | 0.001 | 0.004 | 0.016 | 0.155 | 0.001 | 0.206 | 0.001 | 0.898 | 0.001 | 0.050 | 0.000 |
| **Mn** | 0.74 | 0.30 | 0.70 |  | 0.001 | 0.001 | 0.147 | 7.75 × 10^−6^ | 0.259 | 7.50 × 10^−5^ | 0.389 | 5.57 × 10^−9^ | 0.009 | 6.49 × 10^−6^ |
| **Ni** | 0.51 | 0.26 | 0.56 | 0.61 |  | 0.006 | 0.437 | 0.000 | 0.459 | 0.002 | 0.434 | 0.002 | 0.003 | 6.38 × 10^−8^ |
| **Mo** | 0.38 | 0.43 | 0.48 | 0.60 | 0.53 |  | 0.553 | 0.001 | 0.411 | 0.002 | 0.731 | 0.007 | 0.346 | 0.004 |
| **Se** | 0.04 | −0.15 | −0.30 | −0.30 | −0.16 | −0.12 |  | 0.778 | 0.032 | 0.709 | 0.003 | 0.490 | 0.145 | 0.457 |
| **Co** | 0.74 | 0.37 | 0.60 | 0.77 | 0.68 | 0.61 | −0.06 |  | 0.217 | 1.33 × 10^−6^ | 0.578 | 4.68 × 10^−5^ | 0.001 | 1.64 × 10^−6^ |
| **Pb** | 0.56 | 0.23 | 0.26 | 0.23 | 0.15 | 0.17 | 0.43 | 0.26 |  | 0.026 | 0.011 | 0.022 | 0.003 | 0.146 |
| **Cd** | 0.74 | 0.53 | 0.68 | 0.71 | 0.59 | 0.58 | 0.08 | 0.81 | 0.45 |  | 0.275 | 2.17 × 10^−5^ | 0.001 | 9.75 × 10^−5^ |
| **Hg** | 0.29 | −0.34 | −0.03 | 0.18 | 0.16 | −0.07 | 0.57 | 0.11 | 0.50 | 0.23 |  | 0.088 | 0.004 | 0.332 |
| **Al** | 0.84 | 0.31 | 0.62 | 0.89 | 0.58 | 0.52 | −0.14 | 0.73 | 0.46 | 0.75 | 0.35 |  | 0.002 | 1.96 × 10^−5^ |
| **As** | 0.64 | 0.05 | 0.40 | 0.51 | 0.56 | 0.20 | 0.30 | 0.63 | 0.57 | 0.67 | 0.56 | 0.59 |  | 0.002 |
| **Cr** | 0.71 | 0.36 | 0.69 | 0.78 | 0.86 | 0.55 | −0.15 | 0.80 | 0.30 | 0.71 | 0.20 | 0.75 | 0.58 |  |
